# Lessons learned from two clinical trials on nutritional supplements to reduce aggressive behaviour

**DOI:** 10.1111/jep.13653

**Published:** 2022-01-17

**Authors:** Nienke J. de Bles, David A. A. Gast, Abe J. C. van der Slot, Robert Didden, Albert M. van Hemert, Nathaly Rius‐Ottenheim, Erik J. Giltay

**Affiliations:** ^1^ Department of Psychiatry Leiden University Medical Center Leiden The Netherlands; ^2^ Gemiva‐SVG Group Gouda The Netherlands; ^3^ Behavioural Science Institute Radboud University Nijmegen The Netherlands; ^4^ Trajectum Zwolle The Netherlands

**Keywords:** aggression, lessons learned, multisite, nutritional supplements, trial

## Abstract

**Background:**

Setting up and conducting a randomised controlled trial (RCT) has many challenges—particularly trials that include vulnerable individuals with behavioural problems or who reside in facilities that focus on care as opposed to research. These populations are underrepresented in RCTs.

**Approach:**

In our paper, we describe the challenges and practical lessons learned from two RCTs in two care settings involving long‐stay psychiatric inpatients and people with intellectual disabilities. We describe five main difficulties and how these were overcome: (1) multisite setting, (2) inclusion of vulnerable participants, (3) nutritional supplements and placebos, (4) assessment of behavioural outcomes, and (5) collecting bio samples.

**Conclusions:**

By sharing these practical experiences, we hope to inform other researchers how to optimally design their trials, while avoiding and minimising the difficulties that we encountered, and to facilitate the implementation of a trial. Both trials were registered in the Clinical Trials Register (RCT A: NCT02498106; RCT B: NCT03212092).

## 1 INTRODUCTION

Conducting clinical trials presents many challenges. We performed two pragmatic randomised clinical trials (RCTs) to determine the effectiveness of nutritional supplements to reduce aggression among two populations: (1) psychiatric patients who resided at long‐stay wards within mental health care organisations (RCT A) and (2) people who received care for their intellectual disabilities (IDs; RCT B). We hope that by sharing the difficulties that we encountered and the ways in which we dealt with these challenges, we may help future researchers who want to set up similar trials.

RCTs are considered to provide evidence for the effectiveness of a particular treatment.^1^ Unfortunately, vulnerable individuals with behavioural problems are underrepresented in RCTs, resulting in a lack of evidence‐based care for these groups.^2^ For example, the prescription of antipsychotics as behavioural medication among people with IDs is widespread. However, the evidence for the efficacy of this policy is meagre and has been extrapolated from research on other populations.^3^, ^4^ Another example is the guidelines for treatment of aggression among patients with schizophrenia; these guidelines are based on RCTs whose generalisability is questionable.^5^ Because treatments for aggressive behaviour are used on vulnerable populations within long‐term care, the RCTs should also take place within these populations and settings.^6^

Aggressive behaviour frequently occurs among psychiatric patients^7^ and people with IDs.^8^ A substantial number of individuals admitted to long‐term facilities (e.g., psychiatric patients or people with IDs) may express aggressive behaviours not only as the reason for admission but also as a consequence thereof. Aggressive acts range from mild verbal incidents, such as screaming or swearing, to more severe incidents, such as physical violence and self‐harm.^9^, ^10^, ^11^ The burden of these incidents lies not only with care professionals, often causing distress and sick leave, but also with other admitted individuals and perpetrators themselves.^12^, ^13^, ^14^ There is a need for evidence‐based treatment options to reduce aggression among these populations.

To address this need, we designed two RCTs involving long‐stay psychiatric inpatients and people with IDs. Facilities where these individuals reside, however, generally focus on care rather than research and often have no existing infrastructure to enable clinical research. While conducting the two studies, we encountered many challenges and arranged these into five main topics: (1) multisite setting, (2) inclusion of vulnerable participants, (3) nutritional supplements and placebos, (4) assessment of behavioural outcomes, and (5) collecting bio samples. In this study, we describe the challenges we encountered and how these challenges were overcome.

## 2 MULTISITE SETTING

RCT A and RCT B were set up as multisite randomised double‐blind placebo‐controlled pragmatic intervention studies, which aimed to include 200 participants each. The multisite setting of both trials was necessary to meet their sample size requirement. In addition, a multisite setting features better external validity, which may result in findings that are more generalisable across different settings and circumstances.^15^, ^16^ Despite the advantages, multisite studies tend to be more complicated to conduct compared to single‐site studies. It is necessary to take these complexities into account from the start of the design of a trial.

### 2.1 Challenges and lessons learned

Most sites highly valued both their involvement and our research goals; however, we encountered several barriers, including the recruitment of sites and their internal coordination of the study.

Although it is often hard to recruit participants in regular trials, it can be just as hard (or even harder) to recruit sites.^17^ First, most sites had no research infrastructure. Thus, some personnel were somewhat reluctant to participate because of the anticipated extra workload. Additionally, the reluctance to participate was sometimes caused by reorganisations within some of the sites. As a consequence, we had to approach far more organisations than we had initially anticipated. In our experience, the time it took from our first contact with the organisation to the time the first participants could be included from that site was 1 year (or more). This lengthy process was due to the formal paperwork we needed to obtain the approval by management and the research committees.

Once our sites were recruited, an additional challenge was to involve a coordinator from the institution to help us run the study from the inside. To help us reach our goals, it was important that the inside coordinators had a coordinating function but (more important) that they were also helpful, approachable, and motivated to support the execution of the study—a research champion. A previous study (partially) reimbursed the hours local coordinators spent on the trial.^18^ In RCT B, we did this by paying a fixed amount to a few selected sites for every completed data(set), but doing so did not always lead to higher motivation in local personnel. Another way in which we promoted the engagement of local coordinators was by offering coauthorship when a certain number of participants per site were successfully included. Of course, coauthorship was only possible in cases were the coauthor also made significant scientific contributions to the final manuscript. Unfortunately, this arrangement did not seem to result in a faster or higher recruitment rate.

During the study, we found that consistent personal contact with the care professionals at the sites was essential to maintain motivation. To this end, the RCT A team travelled to the locations every week, and the RCT B team maintained high‐frequency contacts via telephone and e‐mail.

There were three main lessons that we learned from our experiences with the multisite settings. First, recruiting multiple sites is time‐consuming,^17^ which should be taken into account in the time‐management plan of the trial. Second, it is crucial to invest in local coordinators who are intrinsically motivated—often called ‘champions’.^18^, ^19^, ^20^, ^21^, ^22^ And third, frequent (preferably face‐to‐face) contact with the champions and with the other care professionals is important to keep the sites engaged.

## 3 INCLUSION OF VULNERABLE PARTICIPANTS

The inclusion of participants with aggressive behavioural problems poses additional difficulties.^23^ Such individuals tend to be less cooperative and are less likely to be included in clinical trials.^5^ Thus, vulnerable individuals with chronic behavioural problems are often neither willing nor able to give informed consent. We aimed to include participants with psychiatric diagnosis, or ID, who had behavioural problems. Moreover, we also included minors in RCT B, which resulted in extra challenges. In general, individuals who lack the capacity to provide autonomous consent to participate in a study have often been excluded from clinical trials.^2^ Yet, the topic deserves our attention because of the effects on the well‐being of patients themselves, their potential victims, society at large, and the economic costs.^7^, ^24^, ^25^

According to the European Guideline for Good Clinical Practice, researchers are required to give the potential participant a declaration of consent (or ‘informed consent’), which must comply with strict requirements. For instance, the declaration must stipulate the research involved and what the participant is giving permission for (https://english.ccmo.nl/human-subjects/informed-consent). Thus, we had to state the goals of both trials in a manner that suited individuals' level of intellectual ability. For individuals who were (at that moment) incapable of giving consent, a legally authorised representative had to provide consent.

### 3.1 Challenges and lessons learned

During the recruitment, we encountered challenges in recruiting people with aggressive behaviour and in gaining their informed consent.

First, we used the words ‘aggressive behaviour’ in the title of the study and in the information leaflets; as a direct result, many potential participants refused to participate because they did not associate themselves with aggression. To counteract this negative association, we selected a broader and less stigmatising term ‘challenging behaviour’ instead of ‘aggression’ when communicating with the sites in RCT B.

Second, it is important to realise that recruiting vulnerable participants is time‐consuming.^2^ A systematic review of 33 studies on aggressive behaviour in schizophrenia reported a recruitment period of 3 years on average, with a mean sample size of 93.^5^ A main reason is that individuals with aggressive or challenging behaviour generally seem less willing to participate in trials. As a consequence, less aggressive participants were more often included in RCT A than their more aggressive counterparts, leading to a large proportion of participants showing less than three aggression incidents during the trial (46%). Unlike the RCT A trial, participants in the RCT B study were screened for their aggression levels in the run‐in phase of the study and were excluded for randomisation if they did not show an aggression frequency above a certain threshold.

A third challenge was the process of informed consent and how to transfer knowledge to the potential participants, whose cognitive abilities were often poor. For RCT B, we designed an animation (https://www.youtube.com/watch?v=49wDsOYIxsY) to explain the aim of the study in a way that was understandable to participants with mild IDs and borderline intellectual functioning (IQ 50–85). Even so, not all participants had the capacity to provide written informed consent. In RCT A, the treating psychiatrist assessed a patient's capacity. In cases where a participant was unable to give informed consent, a relative or legal representative was needed to give consent (https://english.ccmo.nl/investigators/legal-framework-for-medical-scientific-research/wmo-protection-human-subjects-central/consent). In RCT B, a relative or legal representative had to provide written informed consent in most cases. All procedures complied with the ethical standards of the relevant national and institutional committees on human experimentation and with the Helsinki Declaration of 1975, as revised in 2008. Both trial protocols were approved by the Medical Ethical Committee of the Leiden University Medical Centre (LUMC).

Three main lessons can be learned from our experiences with recruiting vulnerable participants. First, we recommend avoiding the use of potentially stigmatising terms, such as ‘aggression’ or ‘violence’, in the study's description or communication. Researchers in a study on interpersonal violence recommended framing the research question in a nonstigmatizing way (i.e., using a nonthreatening and positive theme).^26^ Second, we advise researchers to screen participants for entry level of the outcome variable to avoid recruiting participants who show little to no aggression; be aware that the recruitment of more aggressive participants is time consuming. Third, researchers should compose information materials tailored to participants with IDs and should including patient representatives and local care professionals during the design phase.

## 4 NUTRITIONAL SUPPLEMENTS AND PLACEBOS

Nutritional supplements are a special type of intervention for RCTs. RCTs require successful blinding, and the choice of the placebo is not as straightforward as it may seem.^27^ To achieve its purpose, a placebo needs to match the sensory characteristics of the active supplements. This includes visual aspects of the capsules (i.e., shape, size, colour, and texture) as well as their weight, taste, and odour. Any sensory differences between the two capsules may impede the blinding.^28^ To address this issue, we used placebo and verum that were made using the same procedures in the same factory (MCO Health for RCT A; Bonusan for RCT B). This resulted in a placebo that was largely indistinguishable from the verum in terms of appearance.

Another aspect in the selection of a nutritional supplement and its placebo concerns the characteristics of the supplements, such as size and shape (swallowability), which can cause participants to drop out of the study.^29^ Therefore, we used soft gel capsules to deliver the supplements in RCT A; gel capsules are known to be relatively easy to swallow and may help to mask unpleasant tastes and odours.^30^ In RCT B, the multivitamin and mineral capsules from the factory were large, and during the preparation of the trial, they proved difficult to swallow for some participants. To increase swallowability, we crushed the tablets and filled the content of a single tablet into two opal‐coloured placebo capsules (Size 0). The 2‐week run‐in phase before randomisation (using placebo capsules) helped us to select participants who were willing and able to swallow the supplements.

### 4.1 Challenges and lessons learned

We encountered several challenges before achieving successful blinding and a low dropout rate.

First, both trials aimed to use indistinguishable supplements, but this was only partly possible. We succeeded in designing similar supplements regarding the visual characteristics (texture and weight); however, the odour and taste of some of the active ingredients proved difficult to mask.^31^ To match the odour of the placebo with the active supplements in RCT B, we added vanilla‐scented silica gel sachets to all of the jars.

A second challenge was that the active ingredients could have caused physical changes in participants, which could have hampered the blinding. One of the most common and unpleasant sensations in trials with omega‐3 is dysgeusia due to the fishy taste.^32^ This could explain why a relatively high percent of participants were able to guess their randomisation group in many previous studies on the effects of fish oil.^33^, ^34^ Furthermore, vitamin B_2_ (riboflavin) may give urine a typical dark yellow/orange colour.^35^ Odour and urine discoloration can be simulated by adding a small amount of riboflavin to the placebo.^36^ Therefore, the RCT B multivitamin placebo contained 10% (0.8 mg) of the riboflavin dose of an active capsule. At the end of RCT B, the participants and care professionals were not able to guess the group assignment above chance level. In RCT A, we did not take these precautions to improve the blinding. As a result, burping was slightly (though significantly) more often reported by participants in the intervention group compared to the placebo group, but the majority of participants and nurses could not guess the condition to which the participants had been assigned.

A third problem concerned swallowability. Problems in swallowing the supplements, combined with characteristics such as odour and taste, could cause participants to drop out from the study.^37^ The dropout rate among children in a previous fish‐oil study ranged from 0% to 58%.^29^ We used a 2‐week run‐in phase to lower such initial dropout rates due to swallowing problems. In addition, we reminded participants in the protocol that the supplements should be taken with meals, which reduced the chance of a fishy aftertaste.^38^ We noticed during the trial that some participants had difficulty taking the supplements daily. During the trial, however, we could not change the intervention. We therefore recommend a feasibility study to test how best to administer the supplement for the specific target population. There are several options besides capsules, such as liquids,^39^ chewable tablets with a tasty flavour,^40^ or food products containing the active ingredients (e.g., drinks, margarine, and eggs), which are also called ‘functional foods’.^41^

Problems concerning blinding methodology and selective dropout are common in diet‐related research.^42^ Based on our experiences, there were three main methods to tackle these problems. First, adding vanilla‐scented silica gel sachets to the jars containing the supplements helped to mask the odour. Second, researchers should select the appropriate supplement form to aid the administration to the target population. Third, researchers should advise participants to take the capsules during their meals. An option that we could have considered was to offer participants a swallowing course.^43^

## 5 ASSESSMENT OF BEHAVIOURAL OUTCOMES

The main objective of both trials was to assess whether nutritional supplementation could reduce aggression incidents. There are different ways to measure aggression, and it is important that the measurement tools are valid and reliable. Both studies defined aggression as ‘any verbal, nonverbal, or physical behaviour that was threatening (to self, others, or property) or physical behaviour that actually did harm (to self, others or property)’.^44^ Aggression can be assessed through self‐ and observer‐rated scales. Most of our participants suffered from limited intellectual and self‐reflective capacities and were therefore less capable of completing self‐report scales accurately. Thus, as a primary outcome, we chose observer‐rated scales, which is the preferred method to investigate state aggression.^45^ In RCT A, we assessed the number of aggression incidents using the Staff Observation Aggression Scale‐Revised (SOAS‐R).^46^ The SOAS‐R is a quick and easy‐to‐use tool and is used in psychiatric settings worldwide.^47^ In RCT B, we assessed aggression using the Modified Overt Aggression Scale (MOAS),^48^ which is used to monitor different types of aggressive behaviours in studies among adults with IDs.^49^

### 5.1 Challenges and lessons learned

There were three main challenges regarding the assessment of behavioural outcomes, including the operational observation of aggression, unreported incidents, and the high turnover of staff.

First, although both trials specified the definition of aggression, care professionals are regularly exposed to aggressive behaviour and may be desensitised to more subtle aggression. It may not be apparent to a seasoned care professional to consider an incident a form of aggression. A problem with measuring aggression in RCT B was that care professionals looked at the objective behaviour as well as the intention of the behaviour. They believed that behaviour without intention to cause harm should *not* be considered aggression. However, intentions are not always clear among participants with IDs, and some behaviour could be a way of seeking attention instead of harming someone (e.g., throwing crockery). During the pretraining, we emphasised that care professionals had to report the objective behaviour, not their interpretation of the behaviour.

Second, we noticed that a worrisome amount of aggression incidents were not documented. We posit two main reasons why these incidents were underreported. First, care professionals may have become hardened by the frequent occurrence of mild to moderate incidents and thus were less likely to report them. Second, care professionals indicated that when their workload increased, sometimes as a consequence of aggression, reporting incidents could be given a lower priority.^50^ These unreported incidents were difficult to monitor in RCT A due to the use of the incident‐based SOAS‐R. When no incidents were recorded during a certain time interval, we could not assess whether this was because no incidents had taken place or because nothing had been reported. In RCT B, we used the time‐based MOAS scale, which allowed us to monitor whether all time intervals were reported.^51^ To reduce underreporting in each trial, research assistants performed weekly monitoring by visiting the local site (RCT A) or by contacting the site via phone or email (RCT B). Because the risk of underreporting is highest for mild‐to‐moderate verbal aggression,^52^ we asked care professionals specifically whether these incidents had occurred since our last contact. If the answer was yes, care professionals were asked to fill in the SOAS‐R or MOAS for that incident.

Last, the high turnover of care professionals was a problem^53^ because information acquired through an observational scale is based on the capacities, experiences, and opinions of care professionals and thus vulnerable to subjectivity and measurement error. To ensure validity and accuracy, we provided (new) care professionals in each study with an interactive SOAS‐R or MOAS training module before the start of the trial to improve accuracy and precision. In RCT B, we even created an e‐learning platform to provide new personnel with a standard form of training.

There are three main lessons to be learned from our experiences regarding the assessment of behavioural outcomes. First, researchers should use a scale that can easily monitor aggressive behaviour and that is validated to assess the outcome in the specific study population. Second, setting up a monitoring plan at regular intervals can help researchers to detect and to reduce underreporting of incidents. Third, we encourage researchers to train (new) care professionals continuously throughout the trial phase to calibrate the assessments of the care professionals.^54^

## 6 COLLECTING BIO SAMPLES

In both studies, we collected a series of bio samples. In RCT A, we collected blood samples to determine compliance. In RCT B, we collected faecal samples to assess the effects on participants' microbiomes.

Biomarkers are more reliable than self‐reports in measuring compliance.^55^ In RCT A, we collected blood samples to measure the concentration of vitamins, minerals, and a fatty acid spectrum, which yielded participants' n‐3 FA levels. We collected two tubes (1 serum separator [SST] and 1 ethylenediaminetetraacetic acid [EDTA] tube) before and after the trial. The blood samples were taken by trained care professionals from the local laboratory appointed to each institution. Most sites had a fixed morning once a week during which blood was collected. In RCT A, the blood samples were sent to the laboratory via regular mail within 24 h. Mailing blood samples offered a cost‐effective approach, which we found to be true in a previous study from our group.^56^ During the two studies in question, we reliably found the essential n‐3 PUFAs in EDTA plasma after next‐working‐day mail delivery. Indeed, vitamins have been shown to be stable after delayed whole‐blood processing among various temperatures and storage time.^57^, ^58^

In RCT B, we took faecal samples before and after the trial to measure the effect of nutritional supplements on the microbiome and to assess whether the changes of the microbiome mediated the effect of nutritional supplements on aggressive behaviour. For the collection of the faecal samples, we developed a sample manual with simple text and images (see Supplementary Material 1). The samples were frozen on site (–20°C), after which the researcher used a small portable freezer to transfer the samples to a –80°C freezer at the LUMC, where they were stored until analysis. Sequencing the 16s rRNA gene is still the most widely used method for gut microbiome analysis because the financial costs are lower than that of whole‐genome metagenomic analysis.^59^ The disadvantage is that the cheaper method provides less information regarding the level of genus, species, and strains, but only of the higher taxa such as family, order, class, and phyla.

### 6.1 Challenges and lessons learned

For biomarkers, there were several challenges regarding the sampling procedure and the transport of the samples. Separate consent was required for the collection of bio samples, but participants who did not consent to donating bio‐samples were still eligible to participate.

First, the blood samples did not always arrive at our laboratory on time, which was due to several reasons. Because of the high turnover of care professionals, the envelope with blood samples (RCT A) was regularly forgotten by care professionals. To reduce this error, it was important to contact care professionals from the site where the participant resided and to communicate directly with the local laboratory. Furthermore, it was important that blood was collected Monday through Thursday because samples needed to be processed on working days. When the baseline samples did not arrive on time, the participant had to postpone the start of the trial and wait for the next opportunity. Belgian institutions could not participate in blood collection because the mail delivery from Belgium took more than 24 h to reach the laboratory.

Second, freezing faeces (RCT B) directly at −20°C regularly resulted in practical challenges because the participants resided at 69 different locations (far more than we had anticipated), and each participant had to produce two samples. Using portable freezers to reach every location was not feasible, so we had to use freezers that were available on site, which was often difficult to arrange. So, even with proper preparation, the logistics of faecal‐sample collection can be complex and time consuming. Therefore, we advise researchers to opt for methods that are straightforward.

Based on our experiences, there were three main lessons in collecting biomarker outcomes. Investing in strong collaboration with the local laboratory and care professionals is important when taking blood samples. When collecting fecal samples, it is essential to distribute a manual that the target group can understand. And when choosing a specific bio sample, it is important to consider the feasibility of the necessary logistics.

## 7 CONCLUSIONS

Conducting a successful RCT among vulnerable populations presents unique challenges, which we have discussed in detail. These trials were conducted in long‐term wards for psychiatric inpatients and people with IDs. Such studies are essential to help develop new evidence‐based treatment options. Facilities where these individuals reside, however, generally focus on care rather than research and often have no existing infrastructure to enable clinical research. Yet, both RCT A and RCT B successfully recruited participants to determine the effectiveness of nutritional supplements to reduce aggression among two different populations. We stumbled upon numerous difficulties and found ways to modify our practices successfully regarding the following aspects: (1) multisite setting, (2) inclusion of vulnerable participants, (3) nutritional supplements and placebos, (4) assessment of behavioural outcomes, and (5) collecting bio samples—all of which were essential for the success of both projects (Table 1). We hope that by sharing our practical experiences, we may enable future researchers to more effectively conduct clinical trials in these populations who could still gain much from improved clinical care.

**Table 1:** Practical recommendations for future research

Topic	Recommendation
1. Multisite setting	Take the recruitment of sites into account in the time‐management plan.
	Choose a key contact within the organisation based not only on that person's function but also someone who is helpful, approachable, and motivated to support the execution of the study.
	Invest time at each site (once a week or more), preferably face to face. Develop a remote consent and enrolment process for situations where face‐to‐face contact is not possible.
2. Recruitment of vulnerable participants	Use subtle terminology. Instead of ‘aggression’ use ‘challenging behaviour’ and other words and phrases with more neutral connotations.
	Screen participants for at least some level of aggression to avoid recruiting participants who show little to no aggression.
	Tailor information materials to participants according to their intellectual abilities and include patient representatives and local care professionals.
3. Nutritional supplements and placebos	Add vanilla‐scented sachets to the jars of supplements.
	Choose an appropriate form of the supplements to aid administration to the target population.
	Advise participants to take the capsules during the meal.
4. Assessment of behavioural outcomes	Use a scale that can easily monitor aggressive behaviour and that is validated to assess the study population.
	Set up a plan to monitor participants at regular intervals to reduce underreporting of incidents.
	Train (new) care professionals continuously throughout the trial phase.
5. Collecting bio samples	Invest in strong collaboration with the local laboratory.
	Develop a simple and illustrated manual that can be understood by the participants.
	If possible, choose a bio‐sample that can be transported reliably and easily to a central laboratory.

## CONFLICT OF INTERESTS

The authors declare that there are no conflict of interests.

## Supporting information

Supporting information.

## Data Availability

The datasets used and/or analysed during this study are available from the corresponding author on reasonable request.
