# Supplementary material for: Lessons learned from two clinical trials on nutritional supplements to reduce aggressive behaviour
Source: J Eval Clin Pract. 2022 Jan 17;28(4):607–14. doi: 10.1111/jep.13653 (PMC9543803; doi:10.1111/jep.13653)
Supplement: Supplementary file 1 — Supporting information. [file JEP-28-607-s001.pdf]

Verzamelen van ontlasting

Hartelijk dank dat je mee wilt werken met het verzamelen van ontlasting.
In deze instructie lees je stap voor stap hoe dat gaat.

1 In het pakket zit een papieren fecesvanger
Vraag aan een begeleider of die de papieren fecesvanger op de wc-bril plakt.
De gebruiksaanwijzingen staan op de fecesvanger.
Een plaatje van de fecesvanger zie je hiernaast.

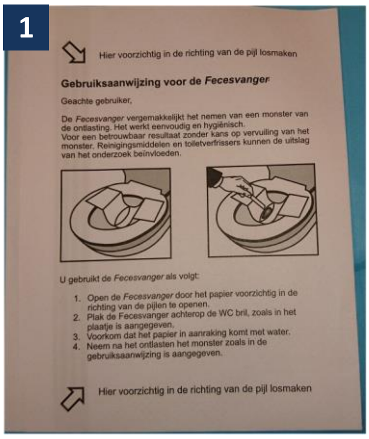
Hier voorzichtig in de richting van de pijl losmaken

Gebruiksaanwijzing voor de *Fecesvanger*

Geachte gebruiker,

De *Fecesvanger* vergemakkelijkt het nemen van een monster van de ontlasting. Het werkt eenvoudig en hygiënisch.
Voor een betrouwbaar resultaat zonder kans op vervuiling van het monster. Reinigingsmiddelen en toiletverfrissers kunnen de uitslag van het onderzoek beïnvloeden.

U gebruikt de *Fecesvanger* als volgt:

1. Open de *Fecesvanger* door het papier voorzichtig in de richting van de pijlen te openen.
2. Plak de Fecesvanger achterop de WC bril, zoals in het plaatje is aangegeven.
3. Voorkom dat het papier in aanraking komt met water.
4. Neem na het ontlasten het monster zoals in de gebruiksaanwijzing is aangegeven.

Hier voorzichtig in de richting van de pijl losmaken

2 Doe je behoefte op de fecesvanger die boven de wc hangt.

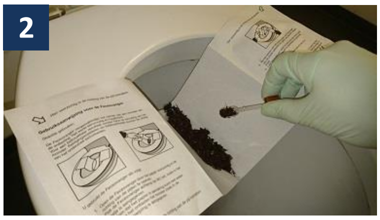

3 In het pakket zitten ook 2 buisjes.
Draai de buisjes open.
Je ziet dat aan het dopje van een buisje een klein schepje zit.
Je haalt een schepje uit het midden van de ontlasting.
Dit doe je voor allebei de buisjes

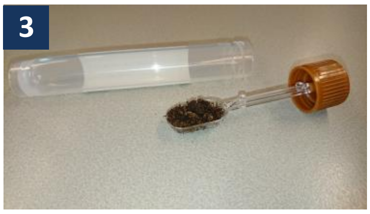

4 Dan doe je het schepje weer in het buisje en draait de dop dicht

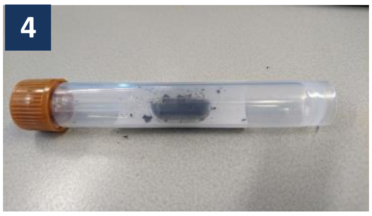

5 In het pakket zit ook een zwart plastic zakje
Je doet de 2 buisjes in het zwarte zakje en klinkt het zakje dicht.

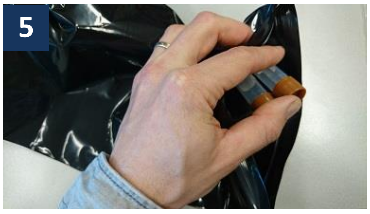

6 In het pakket zit ook een safetybag.
Je doet het zwarte zakje met de buisjes in de safetybag en maakt die dicht.

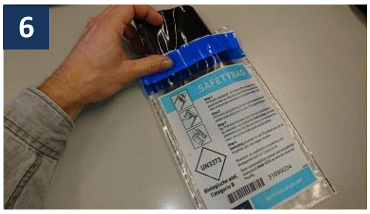

7 Op de achterkant van dit papier staan vragen.
Vul de vragen in.
Als je het niet weet kan de begeleider je helpen.

8 Geef de safetybag met de ingevulde vragenlijst aan de begeleider.
De begeleider zorgt ervoor dat de safetybag binnen 8 uur wordt ingevroren.
De vragenlijst wordt aan de onderzoeker meegegeven.

Hebt u vragen over het verzamelen van ontlasting? Neemt u dan gerust contact op met ons op.
Op werkdagen van 08:00 uur – 17:00 uur bereikbaar via 071 – 526 1517.
Of email: agressievermindering@lumc.nl

Agressievermindering
door supplementen

Deelnemernummer: ____________

Datum: __ __ / __ __ / 2 0 1 __

Hoe laat ging je naar de wc? __ __ : __ __ (uur : min)

Hoe zag de ontlasting eruit? (Bristol Stool Chart)

1	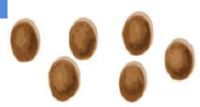	○ 1 Losse harde keutels, zoals noten, moeilijk uit te scheiden
2	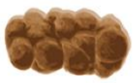	○ 2 Als een worst, maar klonterig
3	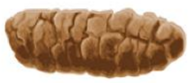	○ 3 Als een worst, maar met barstjes aan de buitenkant
4	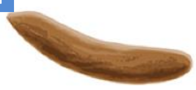	○ 4 Als een worst of slang, glad en zacht
5	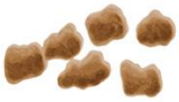	○ 5 Zachte keutels met duidelijke randen, makkelijk uit te scheiden
6	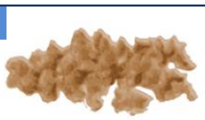	○ 6 Zachte stukjes met gehavende randen, een papperige uitscheiding
7	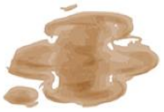	○ 7 Waterig, geen vaste stukjes, helemaal vloeibaar

■ Heeft de dokter een voedselallergie bij je vastgesteld? ○ Nee ○ Ja

■ Heeft de dokter een glutenintolerantie (coeliakie) vastgesteld? ○ Nee ○ Ja

■ Gebruik je momenteel antibiotica? ○ Nee ○ Ja
